# Halide Superionic Conductors for All-Solid-State Batteries: Effects of Synthesis and Composition on Lithium-Ion Conductivity

**DOI:** 10.1021/acsenergylett.4c00317

**Published:** 2024-04-15

**Authors:** Shuhao Yang, Se Young Kim, Guoying Chen

**Affiliations:** Energy Storage and Distributed Resources Division, Lawrence Berkeley National Laboratory, Berkeley, California 94720, United States

## Abstract

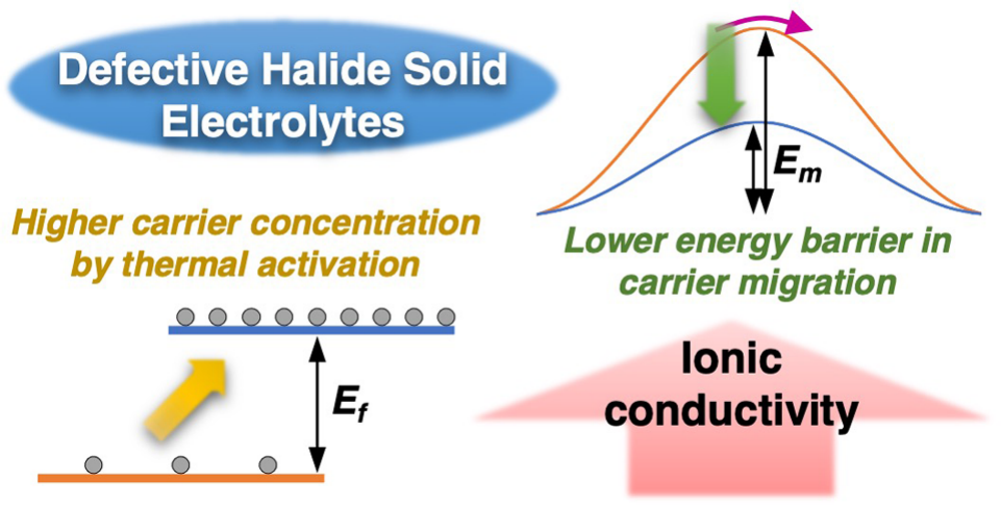

Owing to their high-voltage stabilities, halide superionic conductors such as Li_3_YCl_6_ recently emerged as promising solid electrolyte (SE) materials for all-solid-state batteries (ASSBs). It has been shown that by either introducing off-stoichiometry in solid-state (SS) synthesis or using a mechanochemical (MC) synthesis method the ionic conductivities of Li_3–3*x*_Y_1+*x*_Cl_6_ can increase up to an order of magnitude. The underlying mechanism, however, is unclear. In the present study, we adopt a hopping frequency analysis method of impedance spectra to reveal the correlations in stoichiometry, crystal structure, synthesis conditions, Li^+^ carrier concentrations, hopping migration barriers, and ionic conductivity. We show that unlike the conventional Li_3_YCl_6_ made by SS synthesis, mobile Li^+^ carriers in the defect-containing SS-Li_3–3*x*_Y_1+*x*_Cl_6_ (0 < *x* < 0.17) and MC-Li_3–3*x*_Y_1+*x*_Cl_6_ are generated with an activation energy and their concentration is dependent on temperature. Higher ionic conductivities in these samples arise from a combination of a higher Li^+^ carrier concentration and lower migration energy barriers. A new off-stoichiometric halide (Li_2.61_Y_1.13_Cl_6_) with the highest ionic conductivity (0.47 mS cm^–1^) in the series is discovered, which delivers exceptional cycling performance (∼90% capacity retention after 1000 cycles) in ASSB cells equipped with an uncoated high-energy LiNi_0.8_Mn_0.1_Co_0.1_O_2_ (NMC811) cathode. This work sheds light on the thermal activation process that releases trapped Li^+^ ions in defect-containing halides and provides guidance for the future development of superionic conductors for all-solid-state batteries.

All-solid-state batteries (ASSBs) featuring a lithium metal anode and an inorganic solid electrolyte (SE) have attracted tremendous attention due to their high energy densities and improved safety compared to conventional lithium-ion batteries (LIBs) using liquid electrolytes.^1−3^ Among various SE candidates, newly emerged halide superionic conductors with a general formula of Li_3_MX_6_ (M = Sc, Y, In, Er, Yb; X = Cl, Br) are strong contenders due to their excellent stability at high voltages, enabling the use of 4 V-class cathodes such as LiNi_0.8_Mn_0.1_Co_0.1_O_2_ (NMC811) without a protective coating.^4−9^ However, the ionic conductivities of halide SEs synthesized from the standard solid-state (SS) method are relatively low (<1 mS cm^–1^) compared to other SEs such as sulfides, limiting their application in high-performing ASSBs. To achieve higher ionic conductivities, chemical substitution or mechanochemical (MC) synthesis are often adopted.^10−13^ For halides, substituting trivalent metal cations with tetravalent cations such as Zr^4+^ and Hf^4+^ is widely used,^14−19^ which reduces Li stoichiometry and gives rise to higher ionic conductivities. This is in contrast to the well-known strategy used in inorganic SEs such as LISICON, NASICON, or garnets, where high-valent metal cations are usually replaced by lower-valent ions to increase the Li^+^ content in composition and consequently ionic conductivity.^20−22^ Furthermore, halide SEs synthesized using the MC method are known to have higher ionic conductivity than their counterparts made by the SS method, yet it remains unclear what contributes to these differences.^23−25^

In the Li_3_MX_6_ family, Li_3_YCl_6_ (LYC) and its derivatives (hereafter termed Li–Y–Cl) have shown outstanding chemical/electrochemical stabilities and good compatibility with NMC-type cathodes.^26−29^ While SS-synthesized LYC typically has low conductivities (∼0.02 mS cm^–1^), highly conducting LYC (∼0.4 mS cm^–1^) can be made by MC synthesis. The latter often adopts a disordered trigonal lattice with various defects, which evolves into a more crystalline and ordered structure upon heat treatment. The transformation is often accompanied by a reduction in the ionic conductivity. In addition, the ratio between Li^+^ and M^3+^ was also found to influence the crystal structure and ionic conductivities of halide SEs.^30,31^ An orthorhombic phase of Li–Y–Cl with higher ionic conductivity often forms when the Li stoichiometry is reduced.^32,33^ Note that for common Li–Y–Cl polymorphs, the trigonal and orthorhombic structures are both based on the hcp anion stacking but have different [YCl_6_]^3–^ octahedra arrangements (Figure 1). Both the stoichiometry and synthesis method can greatly affect Li site occupancies in Li–Y–Cl SEs. Analogous to aliovalent substitutions, reducing the Li stoichiometry in Li–Y–Cl SEs leads to lower Li^+^ content in the composition and causes an apparent lower Li^+^ carrier concentration. Although some computational studies suggested that low Li content favors low Li^+^ migration barriers and consequently high ionic conductivities,^34,35^ the effect of Li content on Li^+^ carrier concentration is not fully understood. It is also unclear how MC synthesis affects the concentration and migration of Li^+^ carriers.

**Figure 1:**
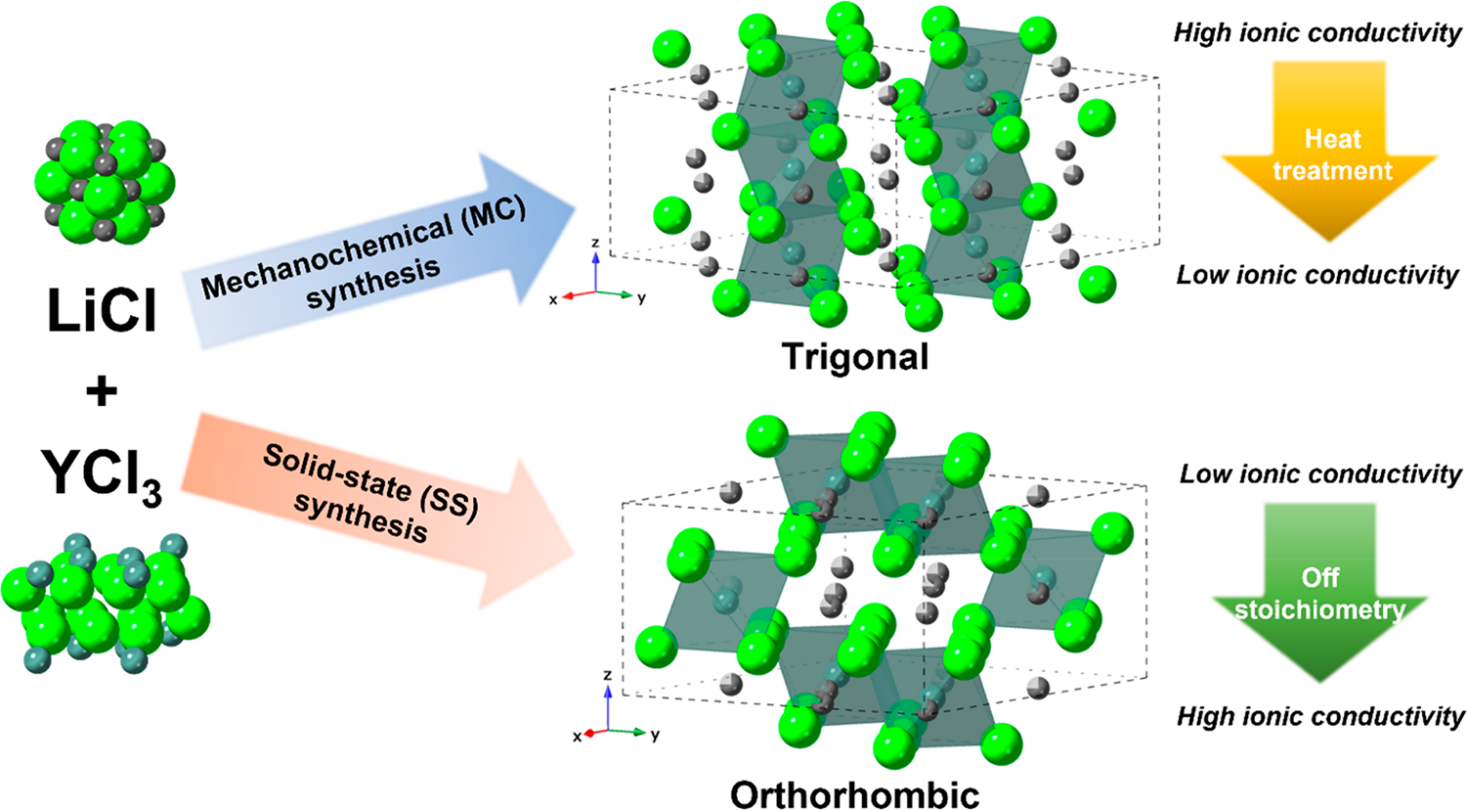
Schematic illustration of synthesis, crystal structure, and ionic conductivities of Li–Y–Cl SEs. The black, blue, and green balls represent Li, Y, and Cl atoms, respectively.

By quantifying the mobility and concentration of mobile ions in ionic conductors, hopping frequency analysis of alternating current (AC) impedance spectroscopy, developed by Almond and West et al. several decades ago, is an effective method for studying carrier behaviors.^36,37^ Combined with variable temperatures, the thermal behavior of ionic conductivity, including formation and migration of carriers, can be evaluated to reveal ion conducting mechanisms in different SEs.^38,39^ In this work, we investigated the effects of MC synthesis and Li stoichiometry on the Li^+^ carriers in Li–Y–Cl SEs using temperature-dependent electrochemical impedance spectroscopy (EIS) measurements. By virtue of hopping frequency analysis, the contributions from the concentration and migration of Li^+^ carriers were deconvoluted and separately determined, revealing a thermally activated mechanism of forming mobile Li^+^ carriers that contribute to ion conduction. MC synthesis and Li-deficient stoichiometry in SS synthesis were found to have similar effects on the Li^+^ carriers, which is associated with the defects in the structure that result in high room-temperature (RT) ionic conductivities. Although previous reports showed structural changes in halide SEs during MC synthesis,^4,6,23−25^ the mechanism of ion transport and ionic conductivity, especially how MC synthesis affects the concentration and migration of Li^+^ carriers, has not been investigated. Our findings not only expand the fundamental understanding of novel structures and their properties achieved through MC synthesis^40^ but also provide new insights on the conduction mechanism that complements the lithium-diffusion kinetics examined by computational methods.^41^ We also examined the electronic conductivity and electrochemical stability of off-stoichiometric Li–Y–Cl SEs and demonstrated the excellent cycling permeance of an NMC811 ASSB cell with a novel off-stoichiometric halide super ionic conductor, Li_2.61_Y_1.13_Cl_6_.

## Synthesis and Crystal Structure

**Figure 2:**
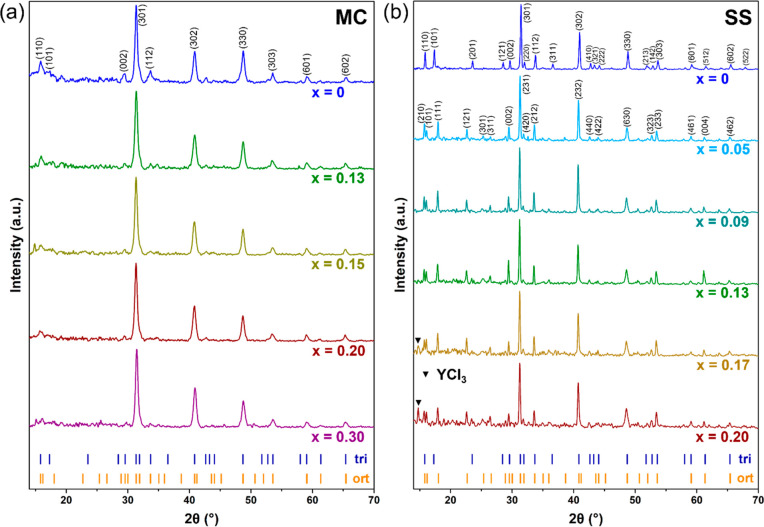
XRD patterns of Li–Y–Cl SEs from (a) MC synthesis (MC-Li_3–3*x*_Y_1+*x*_Cl_6_, 0 ≤ *x* ≤ 0.3) and (b) SS synthesis (SS-Li_3–3*x*_Y_1+*x*_Cl_6_, 0 ≤ *x* ≤ 0.2). Tick marks below the patterns show the peak locations for LYC with the trigonal (tri) and orthorhombic (ort) structures.

By varying the ratio between LiCl and YCl_3_ precursors, we prepared a series of Li–Y–Cl SEs using both MC and SS synthesis. Figure 2 shows the X-ray diffraction (XRD) patterns of the samples. All Li–Y–Cl SEs synthesized by MC have a trigonal phase,^42^ although further structural refinement is difficult due to low peak intensity and the overall low resolution of the XRD patterns (Figure 2a). Despite the broad and overlapping peaks in the XRD patterns, which can be attributed to the low crystallinity of the materials made from MC synthesis,^23^ the selective broadening or even disappearance of specific *hkl* reflections indicates planar defects in the structure.^4^ For example, (101) and (201) peaks are clearly broader or even missing in the XRD patterns of MC-synthesized samples, indicating a high concentration of stacking faults and other defects in these materials.^25^ In contrast, Li–Y–Cl SEs made from SS synthesis have higher crystallinity, and they exhibit sharp peaks in the XRD patterns (Figure 2b). While the stoichiometric SS-LYC can be indexed into the trigonal structure (*P*3̅*m1* space group), a different structure is formed when the Li content is reduced in the composition (SS-Li_3–3*x*_Y_1+*x*_Cl_6_). Based on the splitting peak at ∼16° and the different positions of (111) and (121) reflections compared to those of (101) and (121) reflections for the trigonal structure, SS-Li_3–3*x*_Y_1+*x*_Cl_6_ (*x* > 0) can be indexed into the orthorhombic structure (*Pnma* space group).^43^ The orthorhombic phase exists as a solid solution in a narrow Li-deficient region (0 < *x* < 0.17), and an impurity phase (YCl_3_) starts to appear in SS-Li_3–3*x*_Y_1+*x*_Cl_6_ with *x* ≥ 0.17. All samples show similar morphologies, consisting of agglomerated secondary particles made up of small primary particles several hundred nanometers in size (Figure S1). Owing to the excellent deformability,^4,35^ the particle size differences in the MC and SS synthesized samples were minimized after cold pressing. Both pellets used for the ionic conductivity measurement showed similar morphologies (Figure S2), which allows us to directly evaluate the impact of the structure and composition on the conductivity.

Since the Li_3–3*x*_Y_1+*x*_Cl_6_ (*x* > 0) compounds made by SS synthesis adopt the orthorhombic structure, one may expect off-stoichiometric Li–Y–Cl made from MC synthesis (MC-Li_3–3*x*_Y_1+*x*_Cl_6_) to experience phase transitions from trigonal to orthorhombic upon thermal annealing. In the differential scanning calorimetry (DSC) profiles of MC-Li_2.61_Y_1.13_Cl_6_ (Figure S3), aside from the inverse peritectic reaction (an endothermic peak at ∼480 °C) due to the Li deficiency^44^ and melting of the sample at ∼490 °C, phase transition was not observed. We further conducted heat treatment of MC-synthesized LYC and Li_2.61_Y_1.13_Cl_6_ (MC-LYC and MC-Li_2.61_Y_1.13_Cl_6_) to evaluate the potential phase transition (Figure S4). Heating at 200 °C increases the crystallinity of MC-LYC, and the (101) and (201) peaks are clearly shown in the XRD images after the heat treatment at 400 °C (Figure S4a). However, the trigonal phase remains in the heat-treated samples. For the heated-treated MC-Li_2.61_Y_1.13_Cl_6_, on the other hand, it is difficult to determine the exact phase due to the absence of the peaks corresponding to either (111) and (121) reflections of the trigonal structure or (101) and (111) reflections of the orthorhombic structure (Figure S4b). In all cases, the previously reported metastable β-Li_3_YCl_6_ phase was not observed.^45^

## Ionic Conductivity and Li^+^ Carrier Analysis

**Figure 3:**
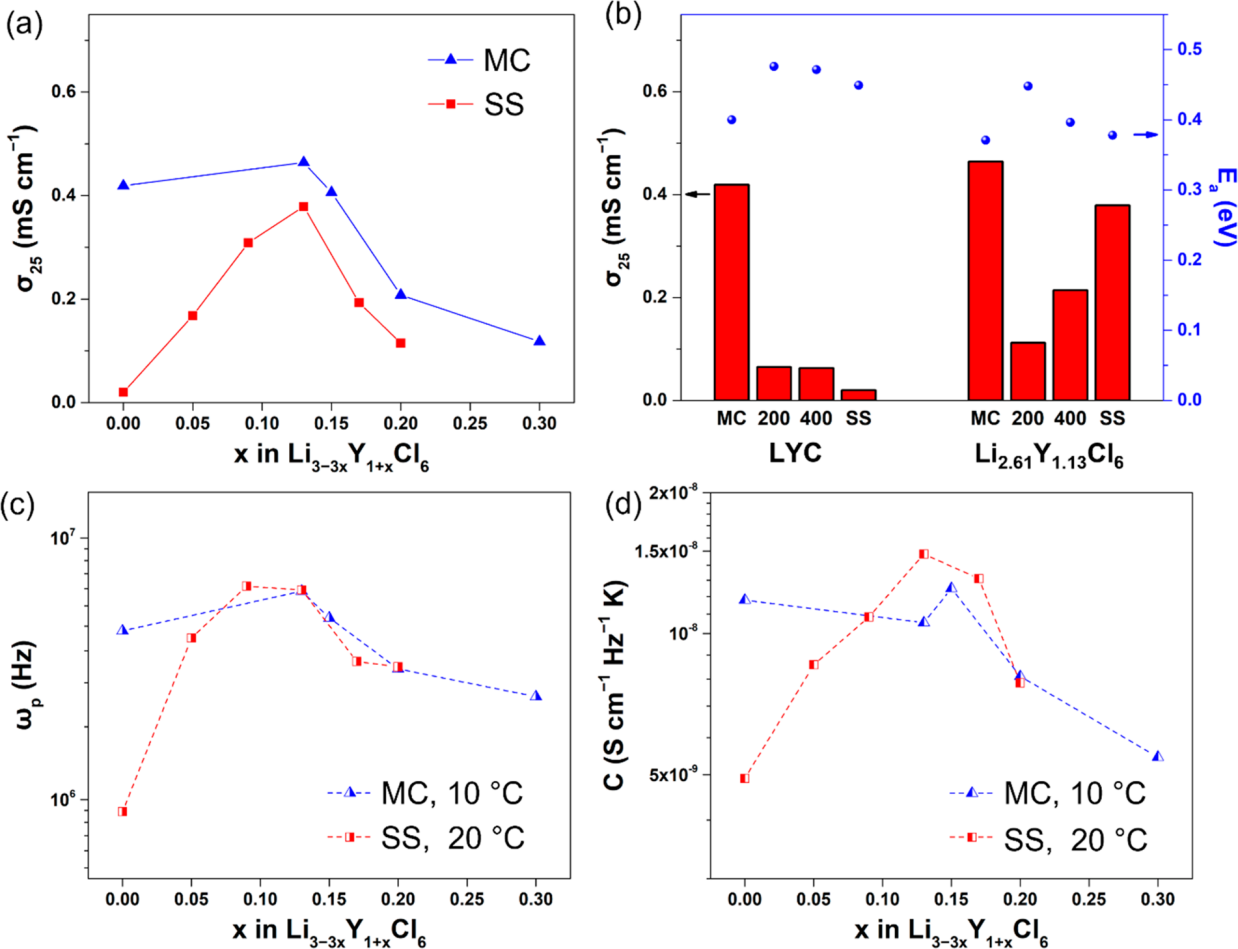
(a) Comparison of ionic conductivities at 25 °C (σ_25_) of Li–Y–Cl SEs from MC and SS synthesis. (b) Comparison of σ_25_ and activation energies (*E*_a_) of as-synthesized and postheat treatment (200 or 400 °C) MC-LYC and MC-Li_2.61_Y_1.13_Cl_6_, and as-synthesized SS-LYC and SS-Li_2.61_Y_1.13_Cl_6_. (c) Hopping frequencies (ω_p_) and (d) carrier concentration factors (*C*) for MC-synthesized Li–Y–Cl SEs at 10 °C and SS-synthesized Li–Y–Cl SEs at 20 °C.

The ionic conductivities of Li–Y–Cl SEs were evaluated by EIS measurements (Figure S5), and the values at 25 °C (σ_25_) are shown in Figure 3a. Orthorhombic SS-Li_3–3*x*_Y_1+*x*_Cl_6_ (0 < *x* ≤ 0.2) show much higher ionic conductivity than that of trigonal SS-LYC (0.02 mS cm^–1^), with the σ_25_ value reaching the maximum of 0.38 mS cm^–1^ for *x* = 0.13. The conductivity decreases upon further increasing the *x* value, likely due to the presence of the ionically insulating YCl_3_ impurity. On the other hand, the ionic conductivities of MC-Li_3–3*x*_Y_1+*x*_Cl_6_ (0 < *x* ≤ 0.15) are similar to that of MC-LYC (0.42 mS cm^–1^). It reaches 0.47 mS cm^–1^ for *x* = 0.13. Beyond that, the σ_25_ value decreases, reaching 0.12 mS cm^–1^ at *x* = 0.3.

The ionic conductivities of the heat-treated samples were also measured by EIS (Figure S6), and the σ_25_ values compared to that of untreated MC and SS samples are shown in Figure 3b. For MC-LYC, the heat treatment decreases the RT ionic conductivity greatly (0.065 mS cm^–1^), which is slightly higher than that of SS-LYC. The decreased ionic conductivities caused by heat treatment are consistent with previously reported results,^23−25^ which was attributed to a decrease in defects/disordering in the structure. The RT ionic conductivity of MC-Li_2.61_Y_1.13_Cl_6_ decreases to 0.112 and 0.214 mS cm^–1^ after heat treatment at 200 and 400 °C, respectively. The higher ionic conductivity at the elevated temperature may be a result of phase transition to the orthorhombic structure,^33^ although no clear evidence was obtained in this study.

To gain insights into the variable ionic conductivities of Li–Y–Cl SEs, especially to separate the contributions from the mobility and concentration of mobile Li^+^ ions, we conducted hopping frequency analysis of the EIS results using the method developed by Almond and West et al.^36−39^ In the AC impedance spectroscopy, the AC conductivity (σ_ω_) is frequency-dependent and has a relationship with the frequency (ω) based on Jonscher’s law of dielectric response^46,47^
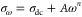
1where σ_dc_ is the direct current (DC) conductivity, *A* is a temperature-dependent parameter, and *n* is the frequency-dependent exponent factor. There is a relationship^48^ between σ_dc_ and *A* in the form of
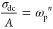
2where ω_p_ is the hopping frequency of mobile ions. By combining eqs 1 and 2, σ_ω_ is given by
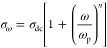
3ω_p_ can be obtained from AC impedance spectra. Figure S7 show the obtained AC impedance spectra and the fitting curves based on eq 3. Note that the highest ω_p_ that can be obtained is limited to <10^7^ Hz due to the frequency limitation of the instrument. We therefore cannot determine ω_p_ in the high-temperature range for some Li–Y–Cl SEs, such as >20 °C for most SS-Li_3–3*x*_Y_1+*x*_Cl_6_ and >10 °C for most MC-Li_3–3*x*_Y_1+*x*_Cl_6_. The ionic conductivity σ (σ_dc_) of any given material has the general expression
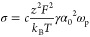
4where *c* is the concentration of mobile ions, *z* is the charge of each ion, *F* is the Faraday constant, *k*_B_ is the Boltzmann constant, *T* is the absolute temperature, γ is the geometric factor, and α_0_ is the hopping distance.^36^ Because factors related to structure (γ and α_0_) are unknown in the studied materials and out of scope of this study, we introduce a carrier concentration factor (*C*) to indicate the relative concentration of Li^+^ carriers, which is defined as
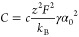
5Then the expression of conductivity becomes
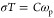
6Once we obtain ω_p_ at a given temperature, the *C* value can be calculated using eq 6. The ω_p_ and *C* at near-RT, i.e., 10 °C for MC-synthesized and 20 °C for SS-synthesized Li–Y–Cl SEs, are listed in Table S1 and shown in Figure 3c,d. Both ω_p_ and *C* values exhibit the same trend as the ionic conductivities at 25 °C. The ω_p_ value for the orthorhombic SS-Li_3–3*x*_Y_1+*x*_Cl_6_ is about an order of magnitude higher than that of trigonal SS-LYC (∼6 × 10^6^ vs ∼9 × 10^5^ Hz), which is comparable to that of MC-synthesized Li–Y–Cl SEs (∼5 × 10^6^ Hz). Among the Li–Y–Cl samples made from SS synthesis, Li deficiency (*x* > 0) increases Li^+^ carrier concentration greatly, evidenced by tripling the *C* value from 4.91 × 10^–9^ S cm^–1^ Hz^−1^ K for *x* = 0 to 1.48 × 10^–8^ S cm^–1^ Hz^−1^ K for *x* = 0.13. Li–Y–Cl SEs from MC synthesis, on the other hand, have relatively constant *C* values (∼1 × 10^–8^ S cm^–1^ Hz^−1^) for samples with various Li stoichiometries.

**Figure 4:**
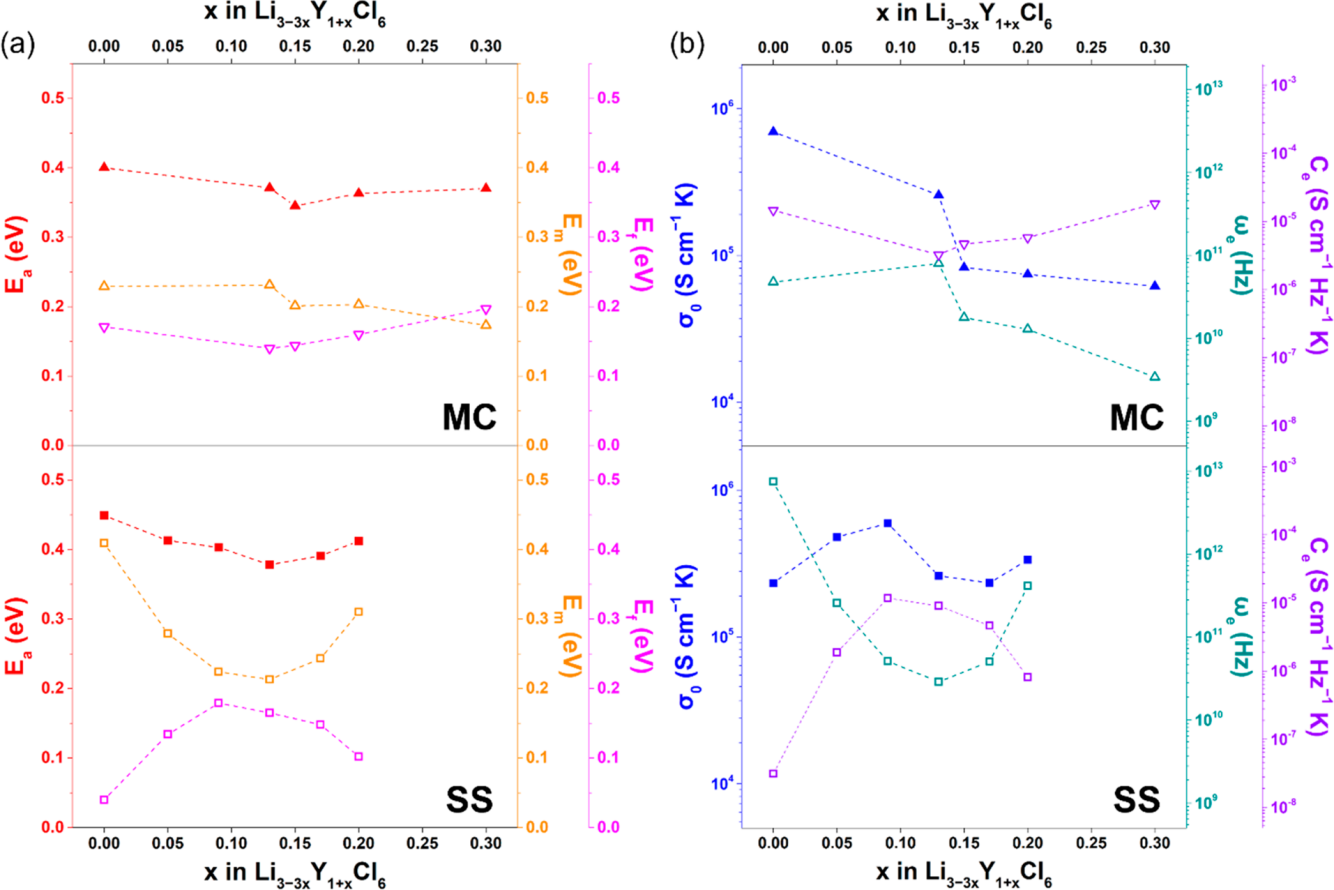
(a) Activation energies of ion conduction (*E*_a_), hopping migration (*E*_m_), and carrier formation (*E*_f_) and (b) Arrhenius prefactors of ion conduction (σ_0_), effective hopping frequencies (ω_e_), and effective carrier concentration prefactors (*C*_e_) for Li–Y–Cl SEs from MC and SS synthesis.

Both ion conduction and hopping migration are thermally activated processes and follow the Arrhenius law:
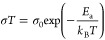
7
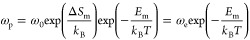
8where σ_0_ and *E*_a_ are the Arrhenius prefactor and the activation energy of ion conduction, ω_0_ is the attempt frequency, *ΔS*_m_ and *E*_m_ refer to the entropy and the activation energy of the hopping migration, and ω_e_ is the effective attempt frequency that includes the entropy term.^37^ By linearly fitting ln(σ*T*) and ln(ω_p_) vs 1/*T* (Figure S8), we obtained the activation energies for ion conduction (*E*_a_) and hopping migration (*E*_m_), respectively (Table S2). Except for SS-LYC, the *E*_a_ values are significantly higher than *E*_m_ values for all Li–Y–Cl SEs (Figure 4a). The differences suggest another component of *E*_a_, which is related to the activation energy of mobile carrier formation (*E*_f_). If mobile carriers are not thermally activated, the concentration of mobile carriers is temperature-independent, and the temperature response of ionic conductivity is only dependent on that of hopping frequency (*E*_a_ = *E*_m_). In such a case, the carrier concentration factor *C* is a constant and the Arrhenius prefactor σ_0_ can be written as
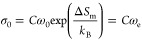
9This applies to SS-LYC which has nearly the same *E*_a_ and *E*_m_ values. On the other hand, if mobile carriers are thermally activated, *C* has the Arrhenius relationship as follows:
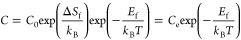
10where *C*_0_ is the carrier concentration factor at infinite temperature, *ΔS*_f_ and *E*_f_ refer to the entropy and the activation energy for the formation of mobile carriers, and *C*_e_ is the effective carrier concentration prefactor including the entropy term. Then, the Arrhenius prefactor σ_0_ in eq 7 has the form
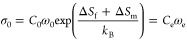
11and *E*_a_ consists of two parts:
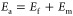
12

Obviously, this scenario fits Li–Y–Cl SEs from MC synthesis or the Li-deficient SS-Li_3–3*x*_Y_1+*x*_Cl_6_. The existence of *E*_f_ reveals that Li^+^ carriers are “trapped” in the lattice that need to be thermally activated to participate in ion conduction.^49^ For the high-energy milling process in MC synthesis and off-stoichiometric composition of SS-Li_3–3*x*_Y_1+*x*_Cl_6_, their crystal structures are expected to be imperfect and they likely contain a large number of defects, such as stacking faults, disordering, and vacancies as suggested in other studies.^23−25,32,33^ On account of the trends of ionic conductivities in Li–Y–Cl SEs, these defects are strongly associated with the fast ion conduction, which includes a thermally activated step to form mobile Li^+^ carriers. In contrast, SS-LYC with a few defects in the structure does not need additional energy to generate Li^+^ carriers but has a much lower carrier concentration. The exponential prefactors in the Arrhenius equations (σ_0_, ω_e_, and *C*_e_) are listed in Table S3 and plotted in Figure 4b. For SS-synthesized Li–Y–Cl SEs, with the decrease in Li content, the Li^+^ carrier concentration becomes more dependent on the thermal activation process along with more trapped Li^+^ carriers in the lattice (as reflected by the increasing *E*_f_ and *C*_e_ values), resulting in the higher concentration of Li^+^ carriers at near-RT (Figure S9). Although the effective attempt frequency (ω_e_) becomes lower as Li stoichiometry decreases (0 < *x* ≤ 0.13 in SS-Li_3–3*x*_Y_1+*x*_Cl_6_), which may be caused by the smaller migration entropy in Li deficient SEs,^50^ the energy barrier that hopping migration needs to overcome (*E*_m_) decreases from 0.409 to 0.213 eV. As a 0.2 eV decrease in *E*_m_ leads to about a two-thousand-fold increase in exp(−*E*_m_/*k*_B_*T*) at RT, much larger than the 2 orders of magnitude difference in ω_e_ of SS-synthesized Li–Y–Cl SEs, the hopping frequency ω_p_ is mainly determined by *E*_m_. When further reducing Li stoichiometry (*x* > 0.13), the observed reverse trends of *E*_f_, *C*_e_, and *E*_f_ values may be attributed to the phase separation and the appearance of the YCl_3_ phase. On the other hand, Li^+^ carriers in Li–Y–Cl SEs from MC synthesis are thermally activated with a considerably large *E*_f_ (∼0.15 eV) to free trapped Li^+^ ions, while the hopping migration needs to overcome a relatively low activation energy *E*_m_ (∼0.2 eV). Varying composition has little effect on the formation and migration of Li^+^ carriers in MC-synthesized Li–Y–Cl SEs, resulting in similar ionic conductivities at RT. Overall, reducing Li stoichiometry in SS synthesis or using MC synthesis have similar effects on Li^+^ carriers in Li–Y–Cl SEs, i.e., introducing more trapped Li^+^ ions that require thermal activation as mobile carriers in lattice and facilitating the Li^+^ hopping migration by lowering the activation energy. As a result of these effects, the RT ionic conductivity is improved.

## Electrochemical Performance

**Figure 5:**
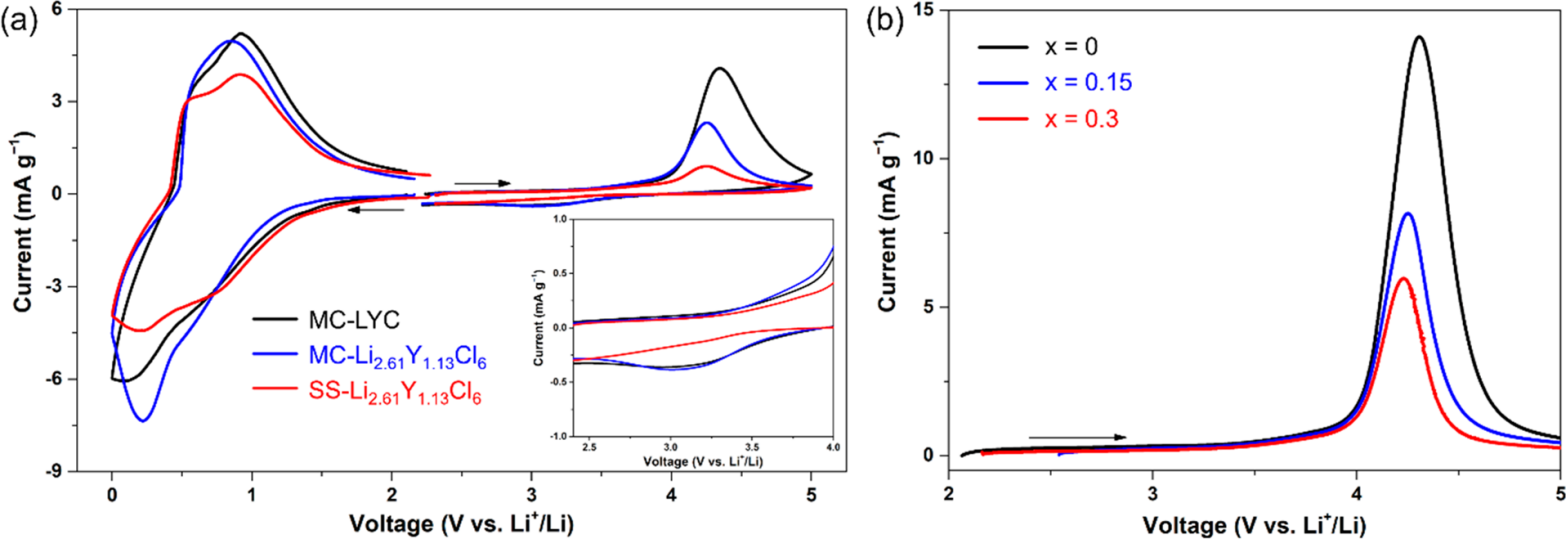
(a) CV profiles of Li–In|SE|SE+C cells with MC-LYC, MC-Li_2.61_Y_1.13_Cl_6_, or SS-Li_2.61_Y_1.13_Cl_6_ as the SE. Inset: an expanded view in the voltage window of 2.5 and 4 V. (b) LSV profiles of Li–In|SE|SE+C cells with MC-Li_3–3*x*_Y_1+*x*_Cl_6_ (*x* = 0, 0.15, and 0.3) as the SE. All scan rates are 0.02 mV s^–1^.

The electronic conductivity and electrochemical stability window of the samples were evaluated by means of DC polarization (Figure S10) and cyclic voltammetry (CV) (Figure 5a), respectively. The measured electronic conductivities of all Li–Y–Cl SEs are extremely low, with 4.39 × 10^–11^, 1.17 × 10^–11^, and 8.54 × 10^–11^ S cm^–1^ for MC-LYC, MC-Li_2.61_Y_1.13_Cl_6_, and SS-Li_2.61_Y_1.13_Cl_6_, respectively. This suggests that the samples are suitable for use as SE separators in ASSB cells.^51,52^ The stability voltage windows of MC-Li_2.61_Y_1.13_Cl_6_ and SS-Li_2.61_Y_1.13_Cl_6_ are similar to that of MC-LYC, with an oxidation onset potential at ∼4.0 V vs Li^+^/Li and two reduction onset potentials at ∼1.2 and ∼0.5 V. The weak redox peak observed at ∼3.3 V during the negative scan may be associated with the reduction process of the oxidation products (e.g., Cl_*x*_^–^) formed at high voltages.^53^ Compared to MC-LYC, Li-deficient MC-Li_2.61_Y_1.13_Cl_6_ shows a smaller anodic (positive) current, which further decreases with a reduction in Li stoichiometry, as shown in the linear sweep voltammetry (LSV) profiles (Figure 5b). These findings suggest that reducing the Li content in the composition may mitigate the degradation of Li–Y–Cl SEs driven by electrochemical oxidation to some degree. Considering the higher ionic conductivity and improved high-voltage stability, Li-deficient Li–Y–Cl SEs can be expected to have a better performance in ASSBs than the stoichiometric LYC.

**Figure 6:**
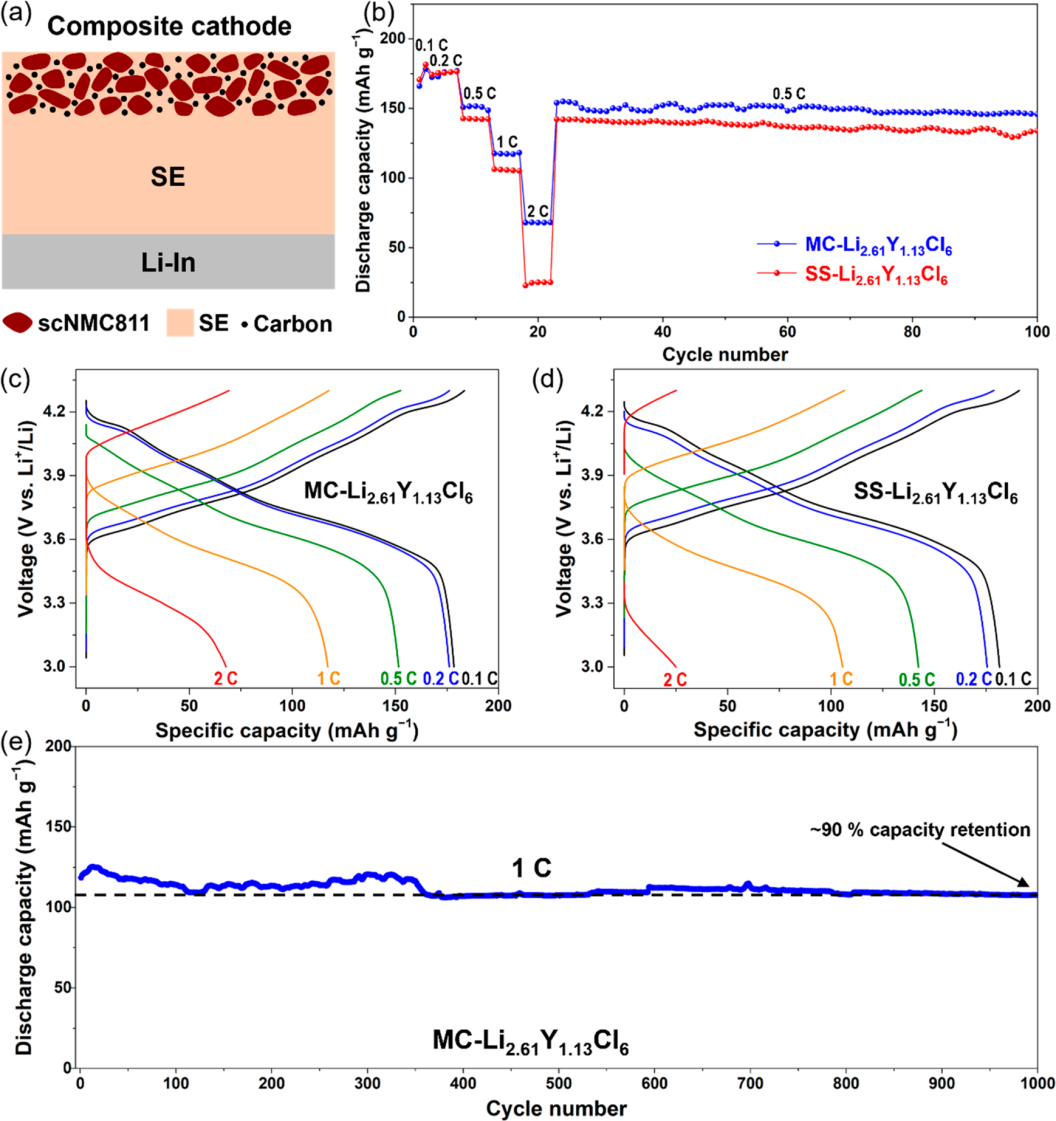
(a) Schematic of the ASSB cell configuration consisting of Li–In|SE|SC-NMC811+SE+C. The weight ratio of NMC811:SE:C is 58:37:5. (b) Room-temperate rate capabilities of MC and SS cells and (c, d) the corresponding charge–discharge voltage profiles at different current densities. (e) Long-term cycling performance of the MC cell at 1 C.

To evaluate the electrochemical performance of off-stoichiometric Li–Y–Cl SEs, we assembled ASSB cells with a single-crystal NMC811 (SC-NMC811) composite cathode, MC-Li_2.61_Y_1.13_Cl_6_ or SS-Li_2.61_Y_1.13_Cl_6_ SE as the separator, and Li–In alloy as the anode (Figure 6a), which are referred to as MC cell and SS cell hereafter. Both cells were cycled at RT in a voltage window of 3.0–4.3 V vs Li^+^/Li, under a constant stacking pressure of ∼8 MPa. It is worth noting that the upper cutoff potential is higher than the stability voltage window indicated by CV. We believe this is mostly due to the interactions between the halide SE and the cathode active materials and the resulting reaction products. Some reactivity between them was previously reported.^27,28^ However, due to the challenges in the characterization of buried interphases, it remains unclear what reaction products are produced and how they can affect the cycling stability. Further studies in this area are required. Another reason could be the low carbon additive content used in the composite cathode (5 wt % vs 30 wt % used in the CV study electrodes). Fewer electronically conducting pathways in composite cathodes are likely to minimize the degradation of the halide SEs.^53^ As shown in Figure 6b, the discharge capacity increases in the initial two cycles at 0.1 *C* (1 *C* = 200 mA g^–1^), which is attributed to a “break-in” process that establishes effective Li^+^ ion migration pathways in the cathode composite.^8^ This process leads to a small charge voltage decay, as shown in the d*Q*/d*V* profiles of the first cycle (Figure S11). At a low current rate of 0.2 *C*, both MC and SS cells delivered a high discharge capacity of 178 mAh g^–1^, which is similar to what was obtained from an equivalent liquid cell. The MC cell showed a better rate performance than the SS cell, delivering specific discharge capacities of 151, 117, and 68 mAh g^–1^ at current rates of 0.5, 1, and 2 *C*, respectively, as compared to 142, 105, and 25 mAh g^–1^ for the SS cell. The improvement may be attributed to the higher SE ionic conductivity in the MC-Li_2.61_Y_1.13_Cl_6_ sample (0.47 vs 0.38 mS cm^–1^ in SS-Li_2.61_Y_1.13_Cl_6_), highlighting the importance of SEs’ ionic conductivity in the performance of ASSBs. EIS spectra of the ASSB cells and the fitted resistance values are shown in Figure S12 and Table S4, respectively. The MC cell had a bulk resistance of the electrolyte (*R*_SE_) that was smaller than that of the SS cell, consistent with the higher ionic conductivity of MC-Li_2.61_Y_1.13_Cl_6_. The resistance in the high- and mid-frequency regions (*R*_HF_ and *R*_MF_) correspond to the grain boundary and cathode/SE interface, while that at the low frequency (*R*_LF_) arises from the anode/SE interface. Both MC and SS cells showed comparable *R*_HF_ and *R*_MF_ values and similar evolution trends during the charge and discharge, further confirming that the performance differences are mostly due to the differences in ionic conductivity. The MC cell was able to sustain ∼150 mAh g^–1^ when the current recovered to 0.5 *C* after the rate capability test. The charge–discharge profiles from 0.1 *C* to 2 *C* are presented in Figure 6c,d, revealing distinct voltage features of NMC811 cathode, including the presence of a high-voltage semiplateau at low rates. The cycling stability of the MC cell is shown in Figure 6e. About 90% of its initial capacity is retained even after 1000 cycles at 1 *C*, demonstrating the excellent cycling stability of MC-Li_2.61_Y_1.13_Cl_6_. For future improvement, we believe it is important to further increase the ionic conductivity of SEs, ideally to be comparable to that of the liquid electrolyte (10 mS cm^–1^). In addition, further optimization of the composition cathode may lead to higher capacity as well as better rate capability.^54,55^

In summary, the ionic conductivity of Li–Y–Cl SEs synthesized from the SS method can be significantly enhanced by reducing Li stoichiometry in the composition or by using an alternative MC synthesis method, both of which introduce defects in the materials. Through hopping frequency analysis of the EIS data, we reveal that the improvement results from the synergetic effect of a higher mobile carrier concentration and lower migration barriers. In both cases, Li^+^ carries are thermally activated and their concentration is temperature-dependent. A new off-stoichiometric Li–Y–Cl SE with a composition of Li_2.61_Y_1.13_Cl_6_ was synthesized using the MC method, which delivered exceptional performance in ASSB cells due to its high ionic conductivity, low electronic conductivity, and good high-voltage stability. A reversible capacity of 180 mAh g^–1^ at 0.2 *C* was achieved, and ∼90% capacity retention after 1000 cycles at 1 *C* was demonstrated. The underlying mechanism revealed in this work, especially the thermal activation process that frees trapped Li^+^ ions in defect-containing materials, offers a new avenue in designing and developing halide superionic conductors as solid electrolytes for all-solid-state batteries.

## Data Availability

The data that support the findings of this study are available in the main text or the Supporting Information of this Letter.

## Supporting Information Available

Experimental methods, SEM images, TG-DSC profiles, XRD patterns of samples, Nyquist plots and DC polarization curves of solid electrolytes, conductivity spectra, Arrhenius plots, carrier concentration factors and other parameters obtained from the hopping frequency analysis, and d*Q*/d*V* profiles and impedance analysis of ASSB cells (PDF)
