# Supplementary material for: Halide Superionic Conductors for All-Solid-State Batteries: Effects of Synthesis and Composition on Lithium-Ion Conductivity
Source: ACS Energy Lett. 2024 Apr 15;9(5):2212–21. doi: 10.1021/acsenergylett.4c00317 (PMC11091881; doi:10.1021/acsenergylett.4c00317)
Supplement: Supplementary file 1 — nz4c00317_si_001.pdf [file nz4c00317_si_001.pdf]

Halide Superionic Conductors for All-Solid-State Batteries: Effects of Synthesis and Composition on Lithium-Ion Conductivity

*Shuhao Yang, Se Young Kim and Guoying Chen**

Energy Storage and Distributed Resources Division, Lawrence Berkeley National Laboratory, Berkeley, California 94720, United States

* Email address: gchen@lbl.gov

Experimental Methods

Materials Synthesis

All samples were synthesized under an inert Ar atmosphere. LiCl (Beantown Chemical, 99.9%) and YCl_3 (Thermo Scientific, 99.99%) were weighed and mixed in different ratios as precursors to synthesize Li-Y-Cl SEs. For MC synthesis, 1.5 g of the precursor was ball milled at 550 rpm for 48 h (a total of 144 cycles of 10 min milling followed by 5 min rest) in a 50 ml ZrO_2 grinding jar with 11 ZrO_2 balls (10 mm) under Ar atmosphere using a planetary ball mill machine (Retsch PM 200). In the middle of ball milling, the jar was opened under Ar and powder stuck to the wall was scraped with a spatula. After the MC synthesis, the product was homogenized in an agate mortar for 15 min by hand grinding. For SS synthesis, 1 g of the precursor was hand-ground together using an agate mortar and pestle for 20 min and then pressed into three 6.5 mm diameter pellets. The pellets were annealed at 450 °C for 24 h (heating rate: 5 °C min^{-1}) and slowly cooled down to RT (1 °C min^{-1}) in a sealed aluminum tube filled with Ar. For heat treatment of MC-

synthesized samples, ~ 0.2 g powder was pressed into a pellet (6.5 mm diameter), annealed at different temperatures in a pre-heated furnace for 2 h in a sealed aluminum tube filled with Ar, and air quenched to RT. No weight changes were observed before and after the annealing process, confirming that the element composition of the products is consistent with the pristine materials. The pellets were hand ground with an agate mortar and pestle for 15 min to get fine powder as the products. All samples were stored in an Ar-filled glovebox without any exposure to ambient air.

Characterization

Powder XRD patterns were obtained using a Bruker D2 Phaser diffractometer operated with Cu Kα radiation (λ = 1.54184 Å) at RT. Samples were transferred to an airtight specimen holder (Bruker) in the glovebox and used for XRD measurements to avoid air exposure. Scanning electron microscope (SEM) images were taken by a field emission SEM (JEOL 7500F) at an accelerating voltage of 15 kV. Thermogravimetry and differential scanning calorimetry (TG-DSC) experiments were performed using a Netzsch STA 449 F1 Jupiter from 30 to 600 °C. ~ 20 mg of the sample was placed in a Pt crucible and heated with a rate of 10 °C min^{-1} under Ar flow (30 mL min^{-1}). A buoyancy correction was performed with an empty crucible prior to the experiment. DSC experiments were conducted using a PerkinElmer DSC 8000 from 30 to 400 °C (heating rate: 5 °C min^{-1}) in a sealed aluminum crucible under Ar.

Conductivity Measurements

Electronic and ionic conductivity measurements were carried out using a custom-built cell consisting of a ZrO_2 mold with 12.7 mm inner diameter and two stainless steel electrodes using a

BioLogic VSP-300 Potentiostat. An SS|SE|SS symmetric cell was assembled by pressing 150 mg of sample uniaxially at 300 MPa for 3 min and a constant stack pressure of ~ 8 MPa was applied during the measurements. The ionic conductivity was determined by AC impedance spectroscopy in a frequency range between 7 MHz to 100 mHz at different temperatures from −20 to 60 °C. An amplitude of 10 mV was applied. The electronic conductivity was determined by DC polarization with an applied voltage from 0.2 to 1.0 V at RT.

Electrochemical Measurements

For electrochemical stability measurements, Li-Y-Cl SEs and carbon black (Super C65) composites in a 7:3 weight ratio were prepared by hand grinding with an agate mortar and pestle for 30 min. Li–In|SE|SE+C cells for cyclic voltammetry and linear sweep voltammetry measurements were fabricated by pressing 30 mg of the Li-Y-Cl SE and carbon composite at 300 MPa on one side of the SE pellet and then attaching Li–In alloy on the other side as the counter electrode. The scan rates for both measurements were 0.02 mV s^{-1}. The cathode composites used in ASSB cells were prepared by grinding SC-NMC811 (Targray), as-synthesized SE, and carbon black (Super C65) with a weight ratio of 58:37:5 in an agate mortar for 30 min. The configuration of the ASSB was a homemade model cell, with solid electrolyte, cathode composite, and Li–In anode pressed into the cell layer by layer using stainless steel terminals and hydraulic press. To assemble the ASSB cell, 100 mg of SE powder was first pressed in a ZrO_2 mold with 12.7 mm diameter under 300 MPa for 1 min. 17.5 mg of the cathode composite (active material mass loading: 8 mg cm^{-2}) was then dispersed on one side of the pelletized SE layer and pressed under 300 MPa for 3 min. A piece of In metal foil (Thermo Scientific, 99.99%, 12.7 mm diameter, 0.127 mm thickness) was placed on the other side of the SE pellet, followed by attaching a piece of Li foil

(Sigma Aldrich, 99.9%) to form a Li–In (3:7 molar ratio) alloy as the anode with a stable potential of 0.62 V vs. Li^+/Li. The stainless steel terminal with a diameter of 12.7 mm (area: 1.267 cm^2) was used as the current collector. The cells were cycled in a voltage window of 2.38–3.68 V vs. Li^+/Li–In (equivalent to 3.0–4.3 V vs. Li^+/Li) under an external pressure of ~ 8 MPa. The processing and stack pressure was calculated based on the force provided by the hydraulic press (YLJ-15, MTI Corporation) and the screw compressing jig (EQ-YLJ-SP, MTI Corporation). Galvanostatic cycling of the cell was carried out using a VMP3 cycler (BioLogic) and rate current density calculations used conventional values for NMC811 (1 C = 200 mA g^{-1}). All electrochemical measurements were carried out at RT.

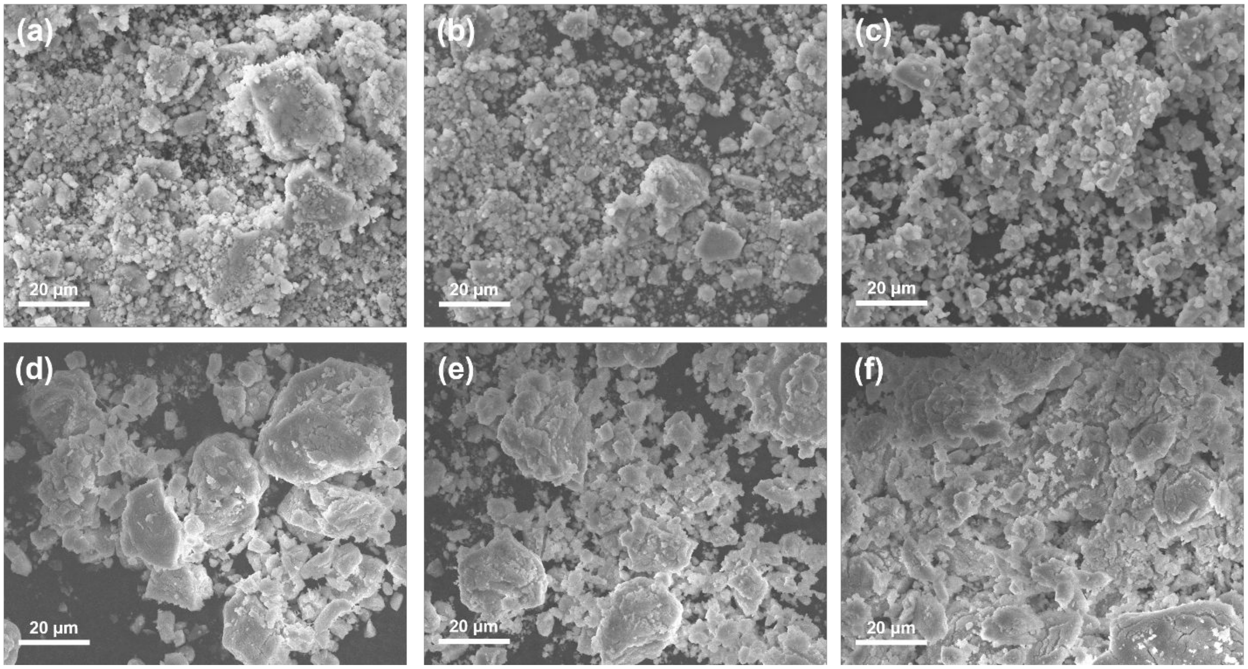

Figure S1. SEM images of (a) MC-LYC, (b) MC-$Li_{2.61}Y_{1.13}Cl_6$, (c) MC-$Li_{2.1}Y_{1.3}Cl_6$, (d) SS-LYC, (e) SS-$Li_{2.61}Y_{1.13}Cl_6$, and (f) SS-$Li_{2.4}Y_{1.2}Cl_6$.

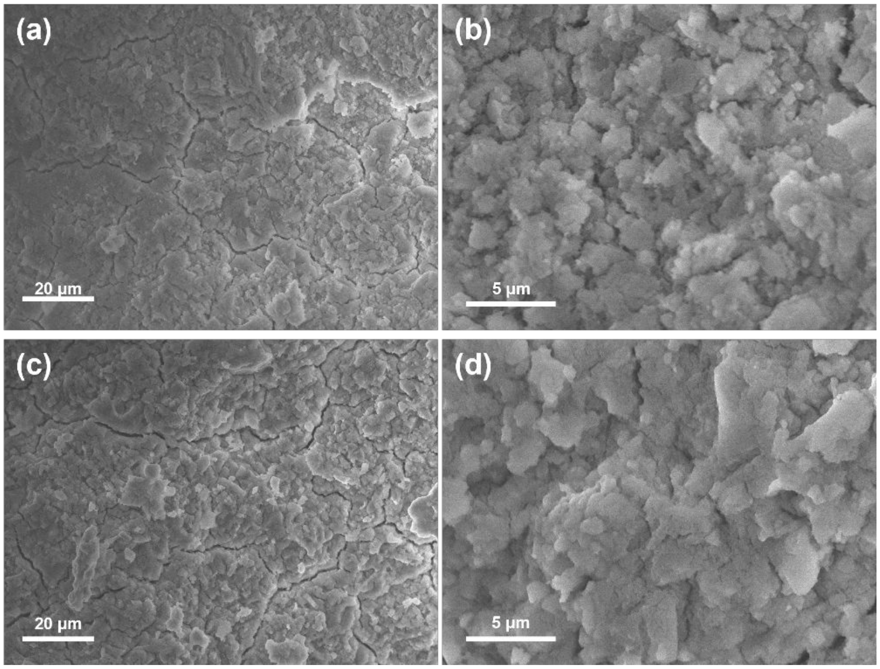

Figure S2. SEM images of cold-pressed (a, b) MC-$Li_{2.61}Y_{1.13}Cl_6$ and (c, d) SS-$Li_{2.61}Y_{1.13}Cl_6$. b) and d) are expanded views of a) and c).

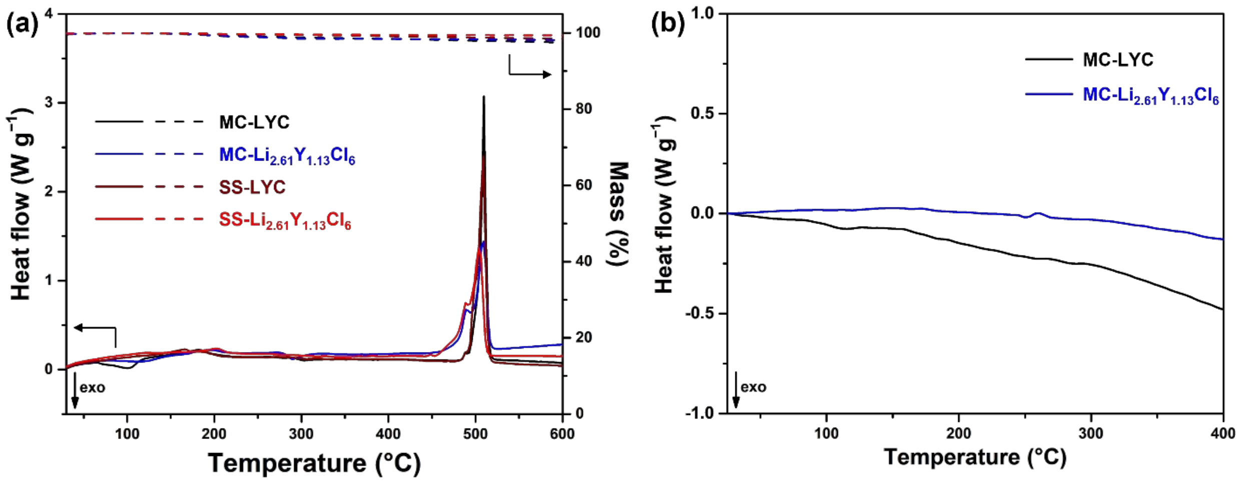

Figure S3. (a) TG-DSC profiles of LYC and $Li_{2.61}Y_{1.13}Cl_6$ obtained from MC and SS synthesis. (Fluctuations of the DSC profiles around 100 °C are due to instrumental artifacts) (b) DSC profiles of MC-LYC and MC-$Li_{2.61}Y_{1.13}Cl_6$ measured from 30 to 400 °C.

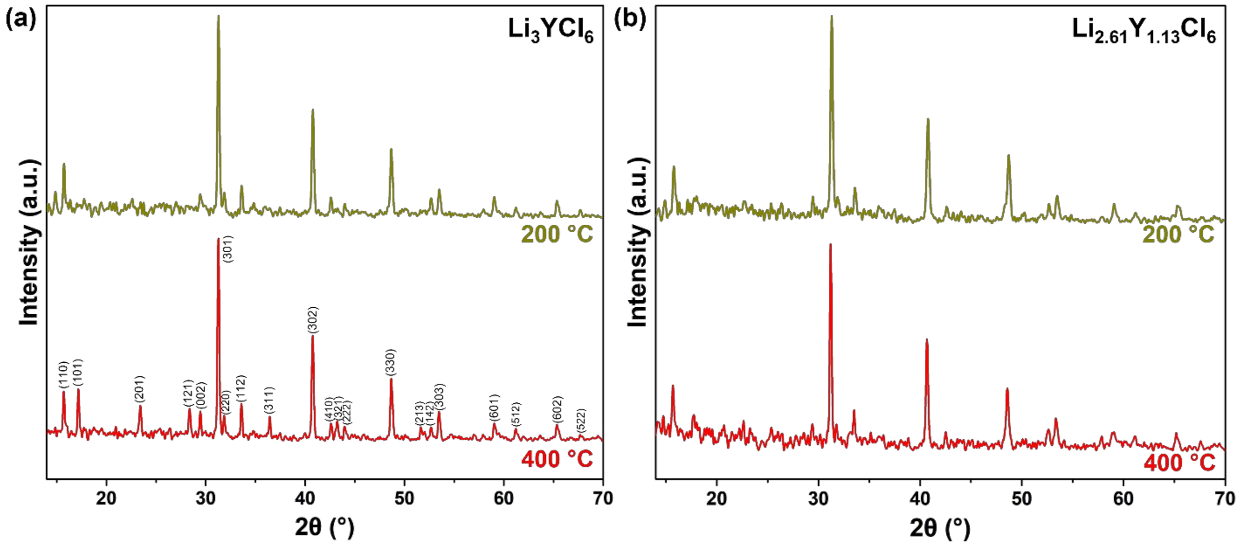

Figure S4. XRD patterns of (a) MC-LYC and (b) MC-$Li_{2.61}Y_{1.13}Cl_6$ after heat treatment at 200 and 400 °C.

a)

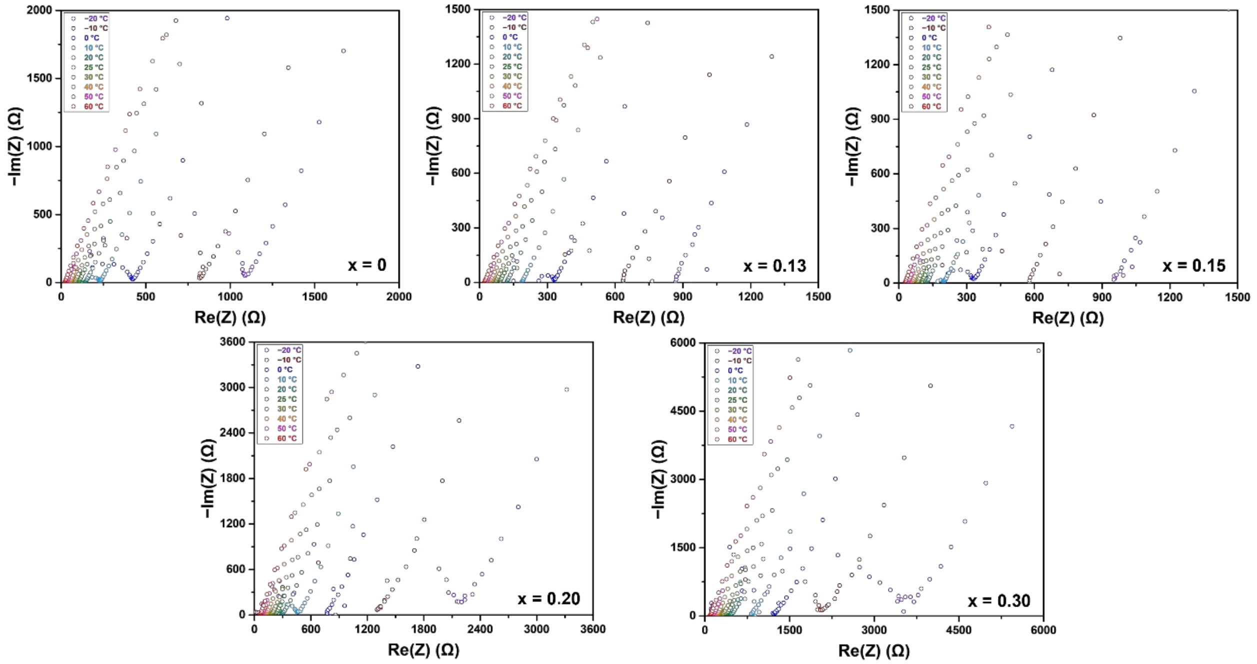

b)

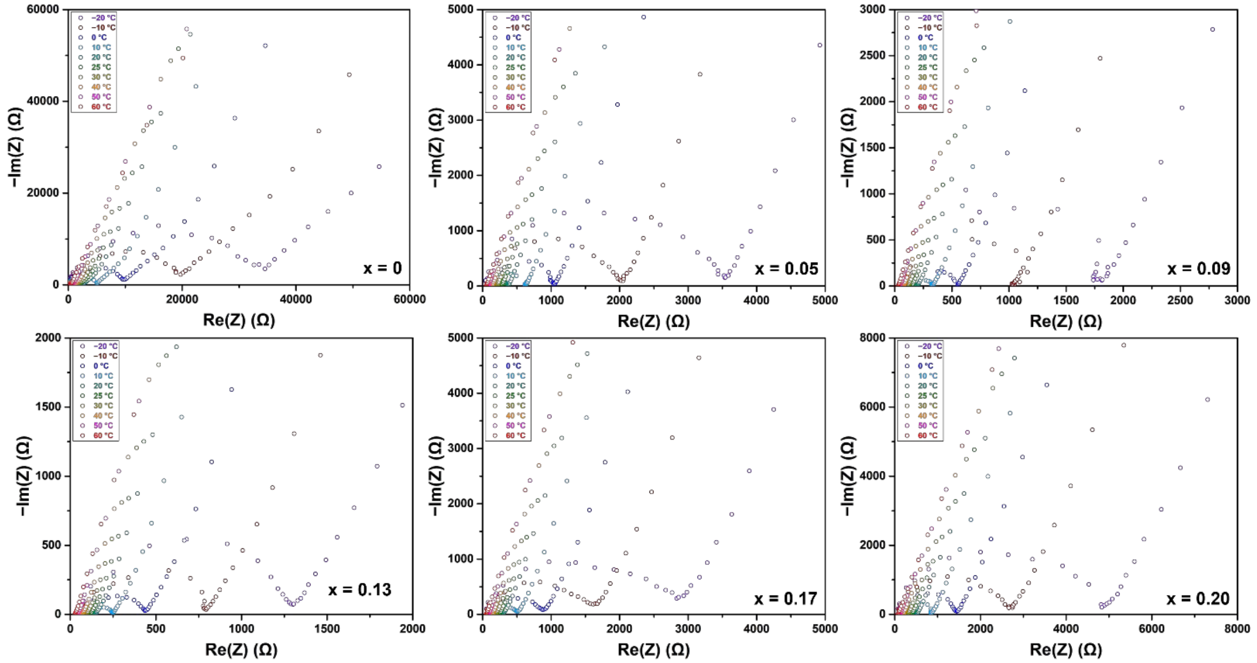

Figure S5. Nyquist plots of a) MC-$Li_{3-3x}Y_{1+x}Cl_6$ ($0 \leq x \leq 0.3$) and b) SS-$Li_{3-3x}Y_{1+x}Cl_6$ ($0 \leq x \leq 0.2$) measured at different temperatures from −20 to 60 °C. x as indicated.

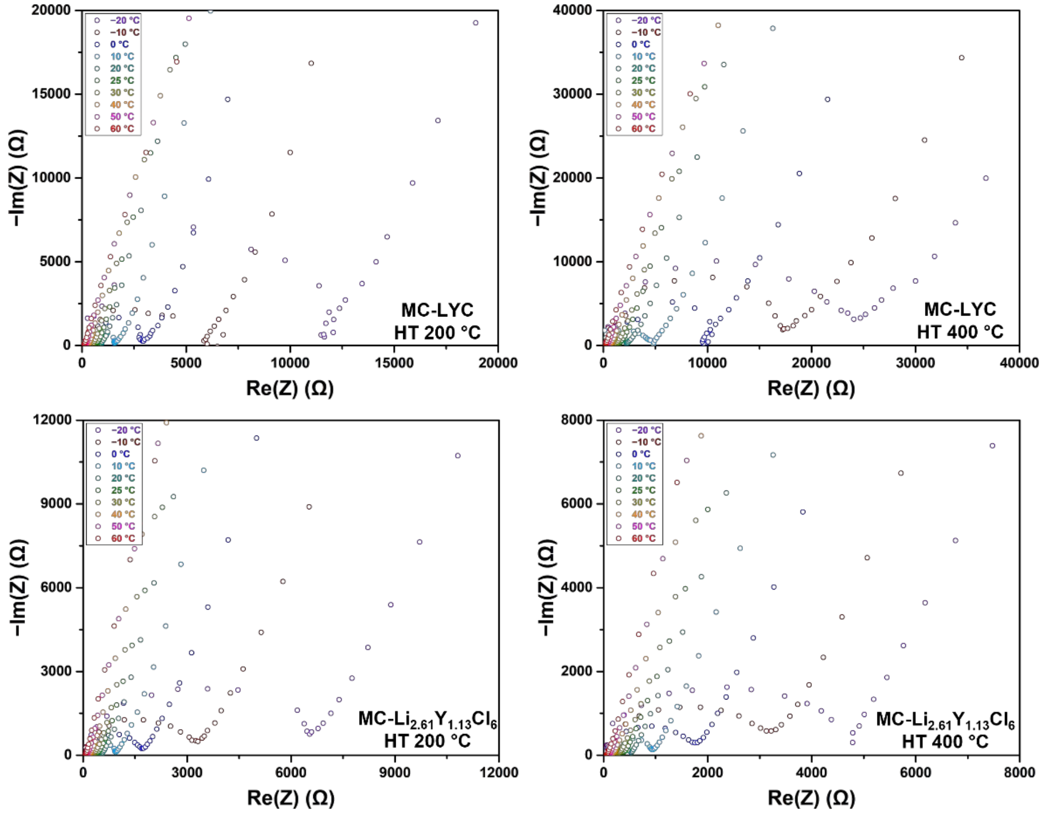

Figure S6. Nyquist plots measured at different temperatures from −20 to 60 °C for MC-LYC and MC-$Li_{2.61}Y_{1.13}Cl_6$ after heat treatment at 200 or 400 °C.

a)

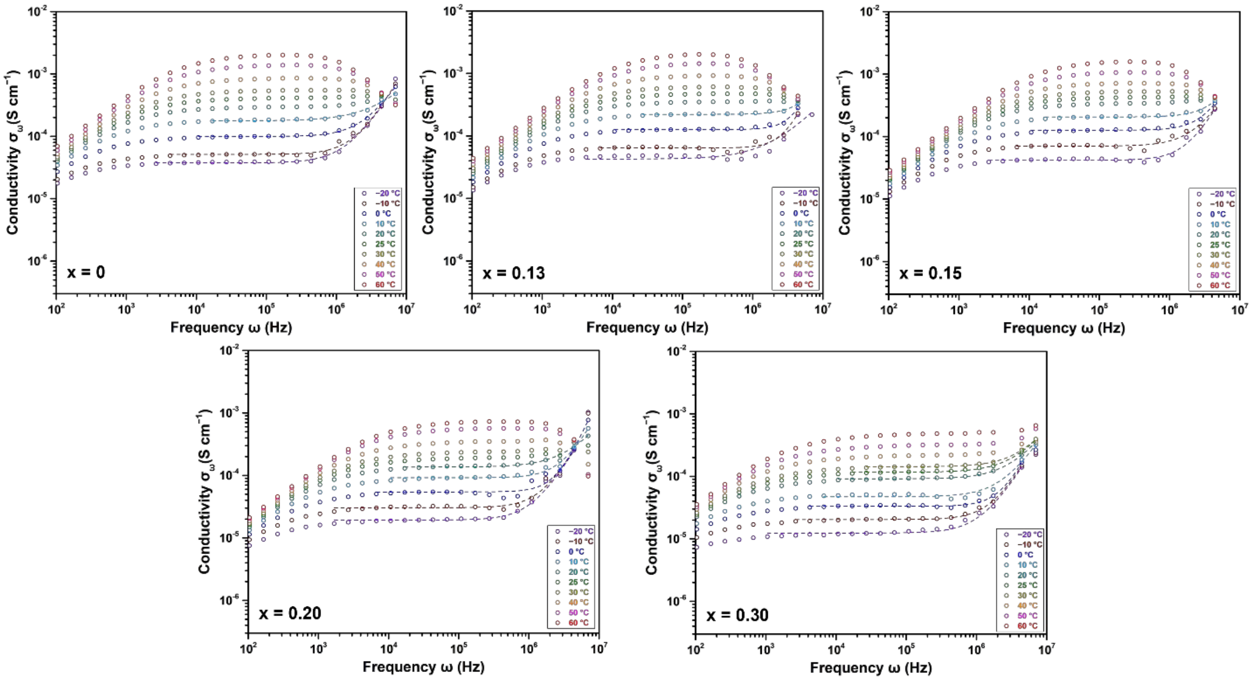

b)

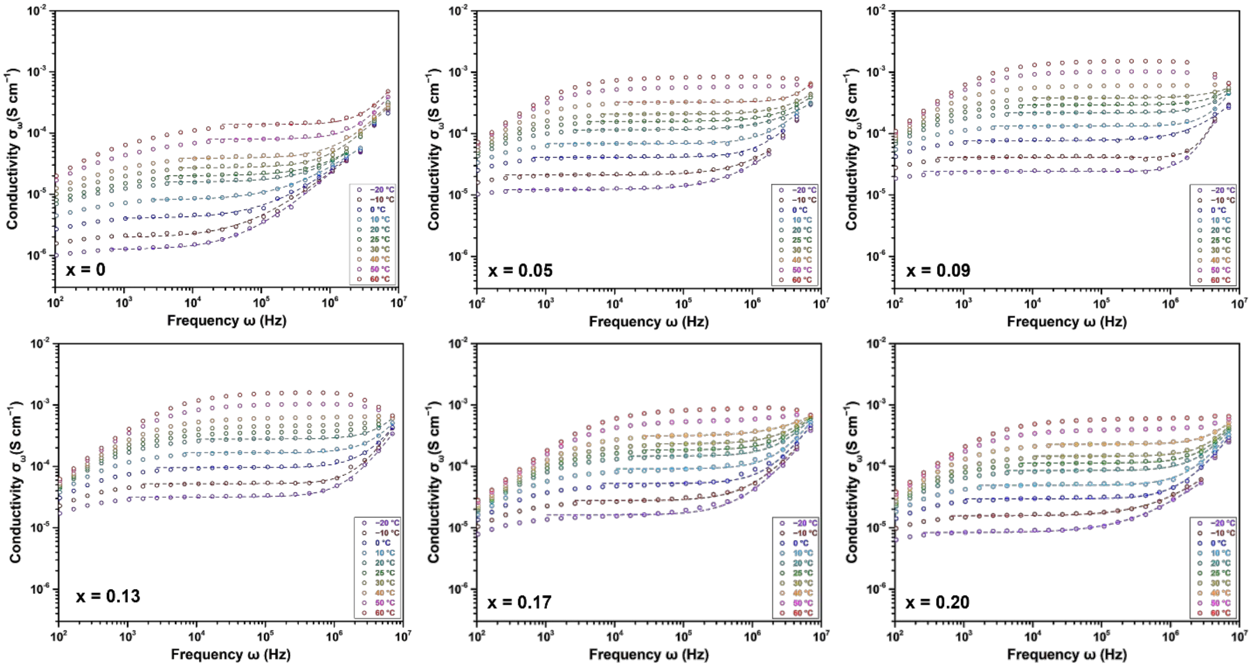

Figure S7. Conductivity spectra of a) MC-$Li_{3-3x}Y_{1+x}Cl_6$ ($0 \le x \le 0.3$) and b) SS-$Li_{3-3x}Y_{1+x}Cl_6$ ($0 \le x \le 0.2$) collected at different temperatures from −20 to 60 °C. x as indicated.

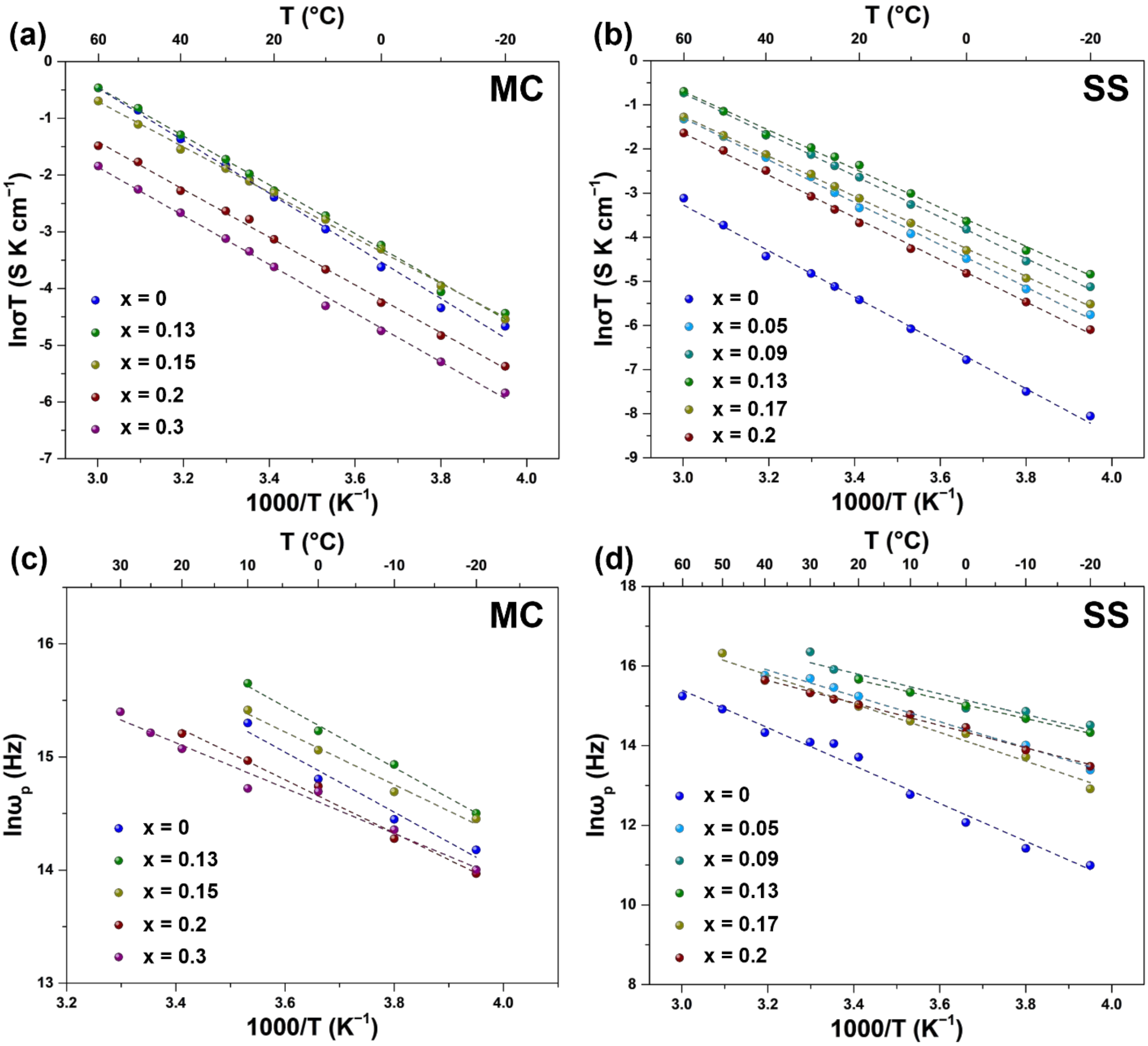

Figure S8. (a, b) Arrhenius plots of conductivity (σT) and (c, d) Arrhenius plots of hopping frequency (ω_p) for MC-$Li_{3-3x}Y_{1+x}Cl_6$ ($0 \leq x \leq 0.3$) and (b, d) SS-$Li_{3-3x}Y_{1+x}Cl_6$ ($0 \leq x \leq 0.2$).

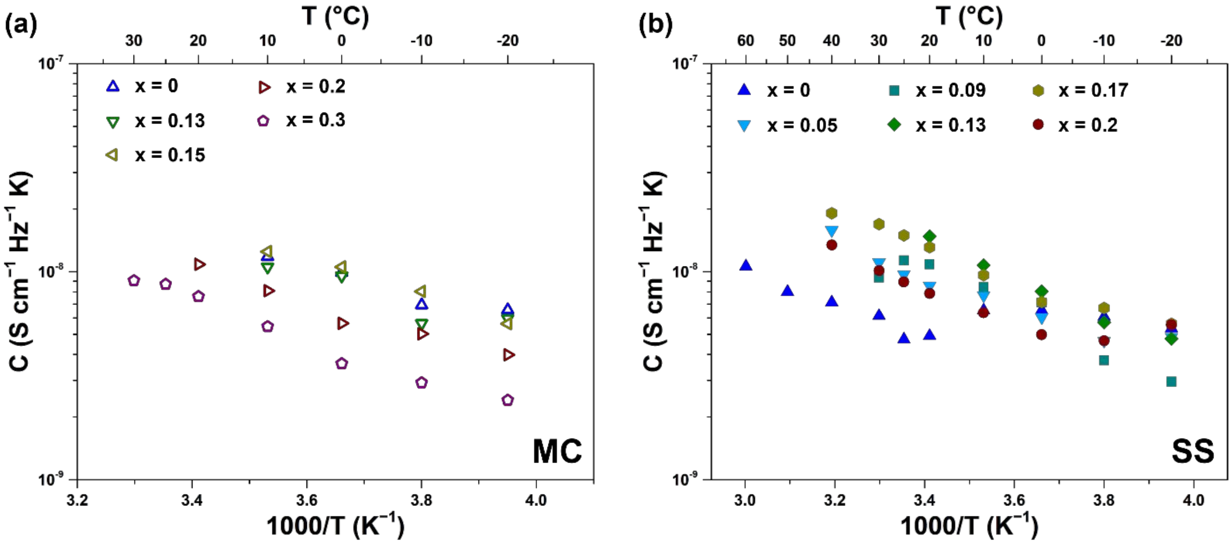

Figure S9. Carrier concentration factors (*C*) at different temperatures for (a) MC-$Li_{3-3x}Y_{1+x}Cl_6$ ($0 \leq x \leq 0.3$) and (b) SS-$Li_{3-3x}Y_{1+x}Cl_6$ ($0 \leq x \leq 0.2$).

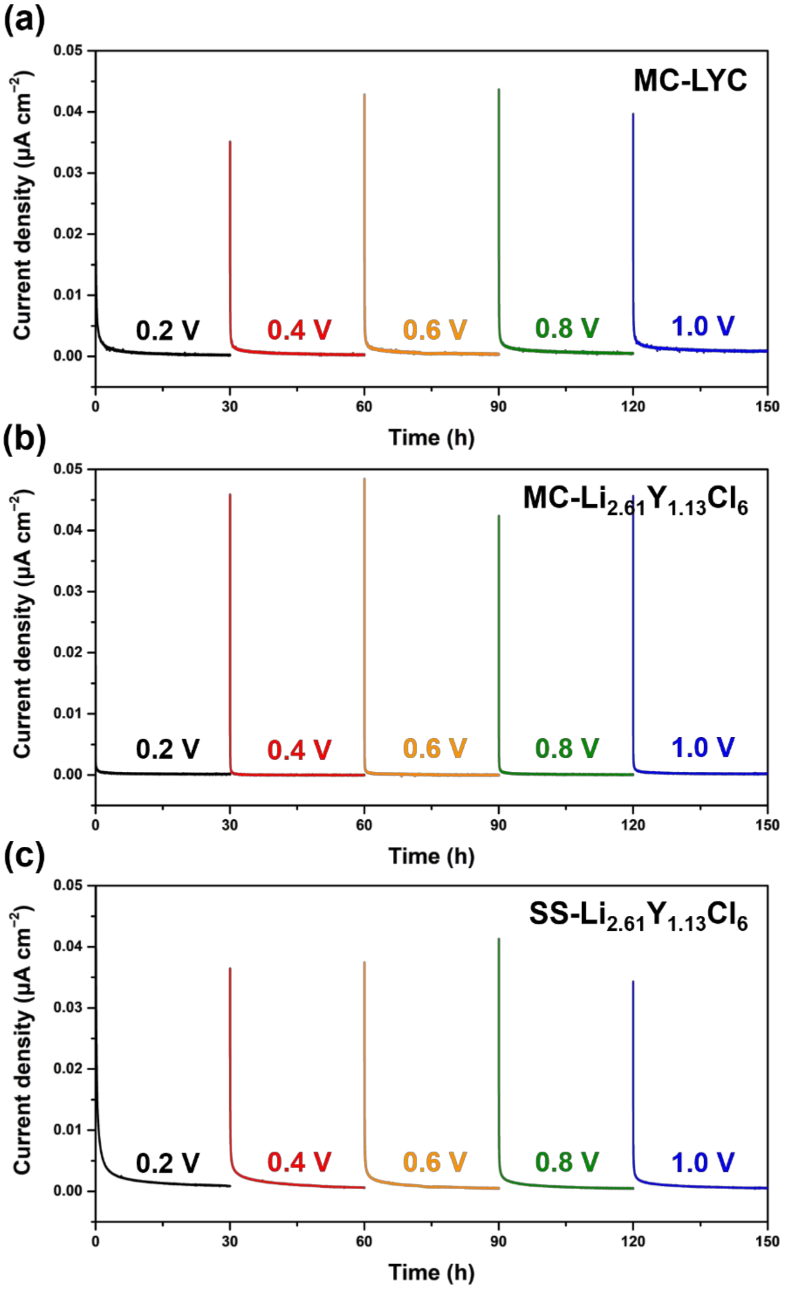

Figure S10. DC polarization curves of (a) MC-LYC, (b) MC-$Li_{2.61}Y_{1.13}Cl_6$ and (c) SS-$Li_{2.61}Y_{1.13}Cl_6$ measured at different voltages from 0.2 to 1.0 V in a symmetric cell configuration with blocking electrodes.

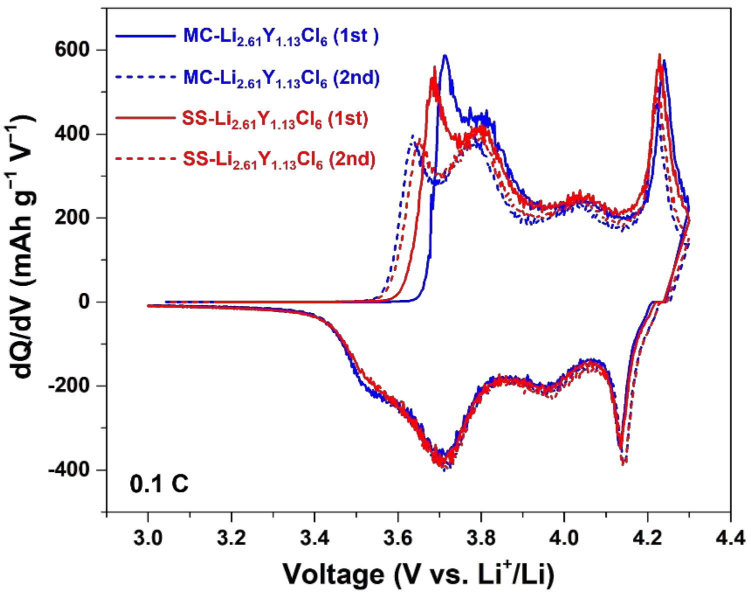

Figure S11. The *dQ/dV* profiles of the first two cycles of Li–In|SE|SC-NMC811+SE+C ASSB cells at 0.1 C. SE is either MC-$Li_{2.61}Y_{1.13}Cl_6$ or SS-$Li_{2.61}Y_{1.13}Cl_6$.

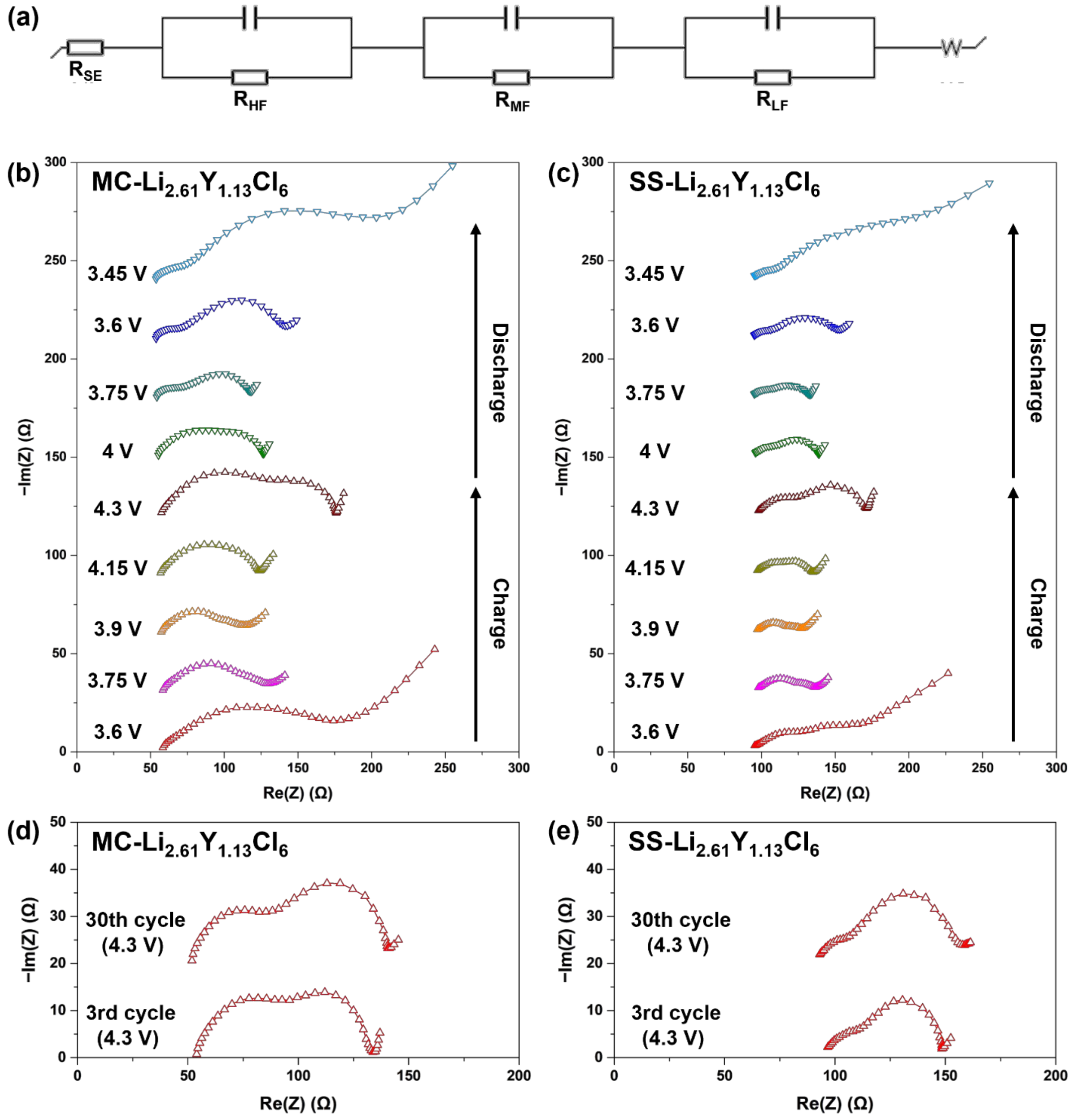

Figure S12. (a) The equivalent circuit used to fit EIS spectra of Li–In/SE/SC-NMC811+SE+C ASSB cells. *In situ* EIS spectra of ASSB cells with (b) MC-$Li_{2.61}Y_{1.13}Cl_6$ or (c) SS-$Li_{2.61}Y_{1.13}Cl_6$ SE during the first cycle at 0.1 C. EIS spectra of ASSB cells with (d) MC-$Li_{2.61}Y_{1.13}Cl_6$ or (e) SS-$Li_{2.61}Y_{1.13}Cl_6$ SE at the charge state (4.3 V vs. Li^+/Li) in the 3rd and 30th cycles at C/3 rate.

Table S1. Ionic conductivities (σ), hopping frequencies (ω_p), and carrier concentration factors (C) for MC-$Li_{3-3x}Y_{1+x}Cl_6$ at 10 °C and SS-$Li_{3-3x}Y_{1+x}Cl_6$ at 20 °C

Synthesis method/Temperature	Sample	σ (S cm^{-1})	ω_p (Hz)	C (S cm^{-1} Hz^{-1} K)
MC/10 °C	x = 0	1.84×10^{-4}	4.41×10^{6}	1.18×10^{-8}
	x = 0.13	2.33×10^{-4}	6.27×10^{6}	1.05×10^{-8}
	x = 0.15	2.18×10^{-4}	4.95×10^{6}	1.25×10^{-8}
	x = 0.20	9.04×10^{-5}	3.16×10^{6}	8.10×10^{-9}
	x = 0.30	4.76×10^{-5}	2.48×10^{6}	5.45×10^{-9}
SS/20 °C	x = 0	1.51×10^{-5}	9.00×10^{5}	4.91×10^{-9}
	x = 0.05	1.21×10^{-4}	4.14×10^{6}	8.57×10^{-9}
	x = 0.09	2.42×10^{-4}	6.54×10^{6}	1.08×10^{-8}
	x = 0.13	3.19×10^{-4}	6.33×10^{6}	1.48×10^{-8}
	x = 0.17	1.51×10^{-4}	3.37×10^{6}	1.31×10^{-8}
	x = 0.20	8.61×10^{-5}	3.22×10^{6}	7.85×10^{-9}

Table S2. Activation energies of ion conduction (E_a), hopping migration (E_m), carrier formation (E_f) for $Li_{3-3x}Y_{1+x}Cl_6$ from MC and SS synthesis

Synthesis method	Sample	E_a (eV)	E_m (eV)	E_f (eV)
MC	x = 0	0.400	0.229	0.171
	x = 0.13	0.371	0.231	0.140
	x = 0.15	0.345	0.201	0.144
	x = 0.20	0.363	0.203	0.160
	x = 0.30	0.370	0.173	0.197
SS	x = 0	0.449	0.409	0.040
	x = 0.05	0.413	0.279	0.134

	x = 0.09	0.403	0.224	0.179
	x = 0.13	0.378	0.213	0.165
	x = 0.17	0.391	0.243	0.148
	x = 0.20	0.412	0.310	0.102

Table S3. Arrhenius prefactors of ion conduction (σ_0), effective hopping frequencies (ω_e), and effective carrier concentration factors (C_e) for $Li_{3-3x}Y_{1+x}Cl_6$ from MC and SS synthesis

Synthesis method	Sample	σ_0 (S cm^{-1} K)	ω_e (Hz)	C_e (S cm^{-1} Hz^{-1} K)
MC	x = 0	6.96×10^5	4.78×10^{10}	1.46×10^{-5}
	x = 0.13	2.57×10^5	7.97×10^{10}	3.23×10^{-6}
	x = 0.15	8.26×10^4	1.78×10^{10}	4.65×10^{-6}
	x = 0.20	7.43×10^4	1.29×10^{10}	5.77×10^{-6}
	x = 0.30	6.17×10^4	3.39×10^9	1.82×10^{-5}
SS	x = 0	2.33×10^5	7.47×10^{12}	3.12×10^{-8}
	x = 0.05	4.80×10^5	2.57×10^{11}	1.87×10^{-6}
	x = 0.09	5.96×10^5	5.10×10^{10}	1.17×10^{-5}
	x = 0.13	2.61×10^5	2.89×10^{10}	9.04×10^{-6}
	x = 0.17	2.34×10^5	5.04×10^{10}	4.63×10^{-6}
	x = 0.20	3.35×10^5	4.12×10^{11}	8.15×10^{-7}

Table S4. Resistance values obtained from fitting the EIS spectra shown in Figure S12

SE	Cycle	Voltage (V vs. Li^+/Li)	R_{SE} (Ω)	R_{HF} (Ω)	R_{MF} (Ω)	R_{LF} (Ω)
MC-$Li_{2.61}Y_{1.13}Cl_6$	1st	3.6	59.8	47.4	44.8	20.9
		3.75	59.6	20.0	31.9	15.1
		3.9	58.6	13.2	15.8	24.1
		4.15	57.9	12.9	22.6	28.4
		4.3	58.9	23.5	45.5	43.0
		4	56.6	15.3	26.1	26.1
		3.75	55.8	13.2	15.6	28.7
		3.6	56.6	17.7	18.4	39.9
		3.45	55.8	18.1	33.3	70.1
	3rd	4.3	56.3	17.1	31.8	23.2
	30th	4.3	53.4	22.5	20.6	40.4
SS-$Li_{2.61}Y_{1.13}Cl_6$	1st	3.6	96.9	29.5	23.0	18.0
		3.75	98.5	9.7	15.4	11.6
		3.9	98.0	9.0	8.3	11.1
		4.15	97.9	9.3	13.4	12.6
		4.3	98.9	18.8	32.8	16.4
		4	96.8	12.2	9.9	18.0
		3.75	96.1	12.5	9.7	12.3
		3.6	96.5	11.9	21.3	18.5
		3.45	97.0	15.4	30.7	45.0
	3rd	4.3	97.9	10.5	25.1	13.5
	30th	4.3	95.2	6.8	16.4	39.9
